# Breast tuberculosis presenting with intractable mastitis: a case report

**DOI:** 10.1186/s13256-021-02712-w

**Published:** 2021-03-04

**Authors:** Yuka Sagara, Shuji Hatakeyama, Ayako Kumabe, Masako Sakuragi, Masami Matsumura

**Affiliations:** 1 Division of General Internal Medicine, Jichi Medical University Hospital, 3311-1 Yakushiji, Shimotsuke-shi, Tochigi, 329-0498 Japan; 2 Division of Infectious Diseases, Jichi Medical University Hospital, 3311-1 Yakushiji, Shimotsuke-shi, Tochigi, 329-0498 Japan; 3 Division of Breast Surgery, Jichi Medical University Hospital, 3311-1 Yakushiji, Shimotsuke-shi, Tochigi, 329-0498 Japan

**Keywords:** Breast tuberculosis, Tuberculous mastitis, Breast cancer, Fistula, Sternal osteomyelitis

## Abstract

**Background:**

Breast tuberculosis, also known as tuberculous mastitis, is an extremely rare form of tuberculosis. It accounts for <0.1% of all breast diseases and <2% of all cases of tuberculosis. It is often misdiagnosed as breast cancer, which can potentially lead to a delayed diagnosis.

**Case presentation:**

A 69-year-old Japanese woman presented with a tumor-mimicking lesion in her right breast, followed by intractable mastitis with a fistula formation. The time until the correct diagnosis of tuberculosis of the breast and sternal bone was 14 months.

**Conclusions:**

Although rare, it is important to recognize that tuberculous mastitis can present as refractory abscesses/mastitis or mass lesions that mimic carcinomas in women of reproductive age and elderly people. Breast tuberculosis should always be considered in the differential diagnoses, particularly in patients with a history of tuberculosis and those living in areas where tuberculosis is endemic.

## Background

Infectious mastitis is common in young lactating women, and the major causative bacteria are *Staphylococcus aureus* and *Staphylococcus epidermidis* [1]. Periductal mastitis and idiopathic granulomatous mastitis are known as non-lactational mastitis. Periductal mastitis has been reported to be related to a history of smoking and infections caused by anaerobic bacteria, staphylococci, enterococci, and *Proteus* spp. [2]. Idiopathic granulomatous mastitis might be associated with *Corynebacterium kroppenstedtii* infections [3].

*Mycobacterium tuberculosis* is a rare but important causative organism of mastitis, especially in chronic or intractable cases. Breast tuberculosis is difficult to diagnose and is often misdiagnosed as breast cancer. We report here a case of tuberculous mastitis that presented with a thoracic mass, breast fistula, and sternal osteomyelitis. The patient underwent various examinations and was treated for mastitis with no improvement, and the correct diagnosis was made at 14 months. Written informed consent was obtained from the patient for publishing the case report and images.

## Case presentation

Fourteen months prior to the referral to our institution, a 69-year-old Japanese woman presented at an orthopedic hospital with a painless mass, approximately 10 mm in diameter, in the right breast adjacent to the sternal border. Based on contrast-enhanced computed tomography (CT) findings, the breast mass was tentatively diagnosed as a benign tumor. However, the lesion gradually increased in size. Four months later, she was referred to the breast surgery department of a community hospital. Ultrasound imaging showed a 17 mm mass surrounded by fluid in the right breast. Cytology of the needle biopsy specimens revealed nonspecific inflammatory cells without atypical cells. Bacterial cultures of the specimens yielded no pathogens. She was administered cefcapene pivoxil 100 mg three times a day for 12 days; however, there was no improvement. She was diagnosed with nonspecific (non-purulent) mastitis, and incisional drainage of the lesion was performed.

Eight months later, the breast lesion, which still continued to increase in size, spontaneously discharged pus from the skin around the right nipple. The patient was referred to the breast surgery department at our hospital, and further referred to our department for the treatment of intractable mastitis.

Her medical history revealed miliary tuberculosis at the age of 25 years, for which she had been hospitalized and treated with three drugs, including streptomycin, for 1 year. She had been a homemaker since her twenties, living with her husband. Her socioeconomic status was not low. She had five pregnancies, three abortions, and two live births. She was an ex-smoker with a smoking history of 42 pack-years. Currently, she was taking clopidogrel and aspirin for ischemic heart disease, bisoprolol and enalapril for hypertension, and lovastatin for dyslipidemia. Her family history was unremarkable.

**Fig. 1.**
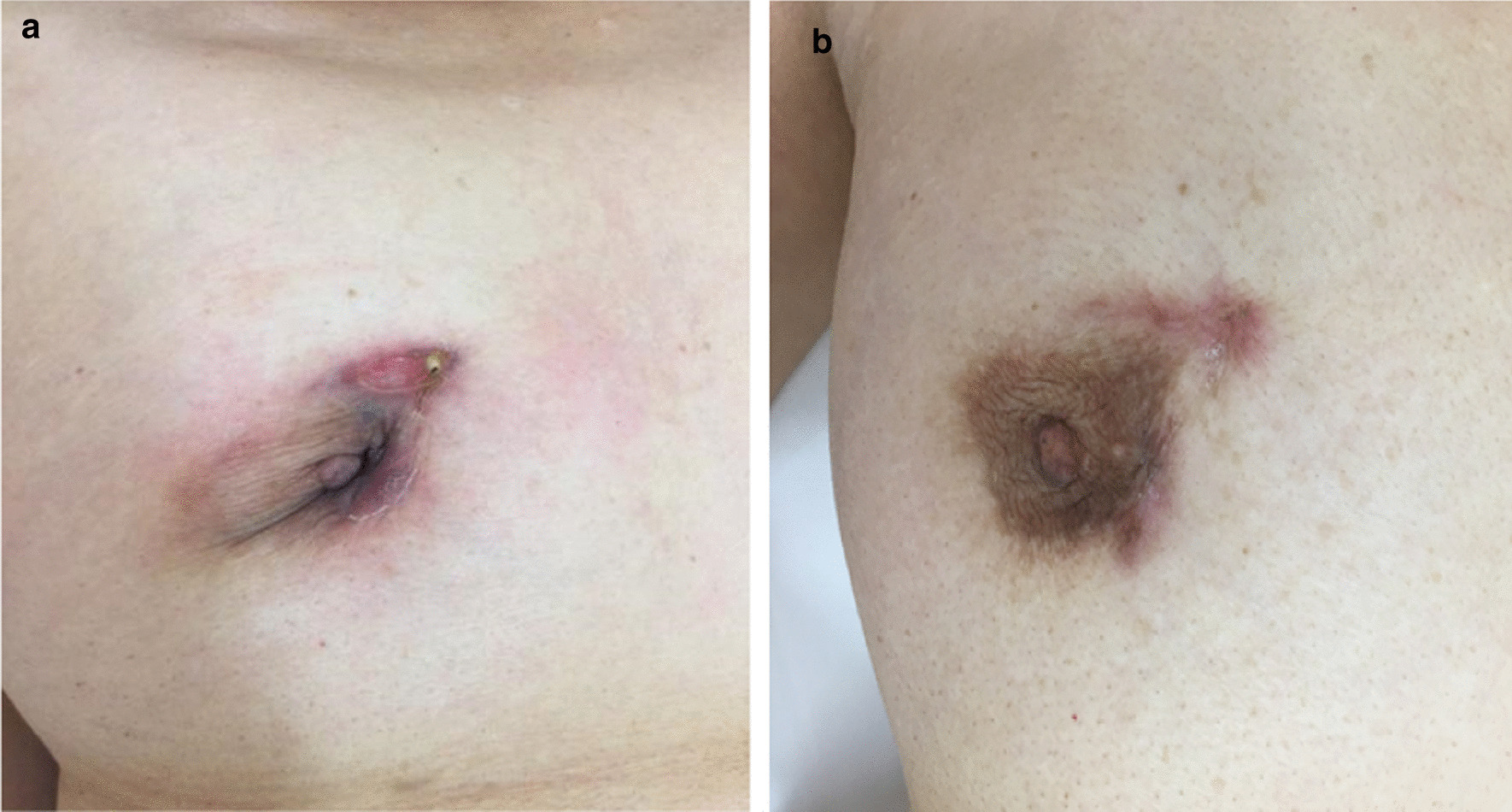
Appearance of tuberculous mastitis. **a** Before treatment: a fistula with persistent pus discharge and retraction of the right nipple. **b** After completion of anti-tuberculosis treatment: closed fistula and improvement of the deformities in the nipple and areola

On examination, she appeared well with stable vital signs: body temperature, 36.7°C; blood pressure, 144/76 mm Hg; pulse rate, 96 beats per minute; and respiratory rate, 14 breaths per minute. The cervical and axillary lymph nodes were not palpable. Heart sounds were normal without murmurs, and the lung sounds were clear without crackles. Her abdomen was soft and flat with no hepatosplenomegaly. Clinical breast examination revealed palpable cord-like induration with mild tenderness from the right margin of the sternum to the skin around the right nipple. The formation of a fistula was observed near the right nipple, and it showed slightly cloudy and yellowish exudates (Fig. 1a).

The laboratory test results were as follows: white blood cell count, 5200/µL; hemoglobin, 11.9 g/dL; platelet count, 159,000/µL; albumin, 4.5 g/dL; aspartate aminotransferase, 21 U/L; creatinine, 0.65 mg/dL; and C-reactive protein, 0.08 mg/dL (reference: < 0.06 mg/dL). Interferon-gamma release assay (T-SPOT.TB^®^) showed positive results. She tested negative for human immunodeficiency virus.

**Fig. 2.**
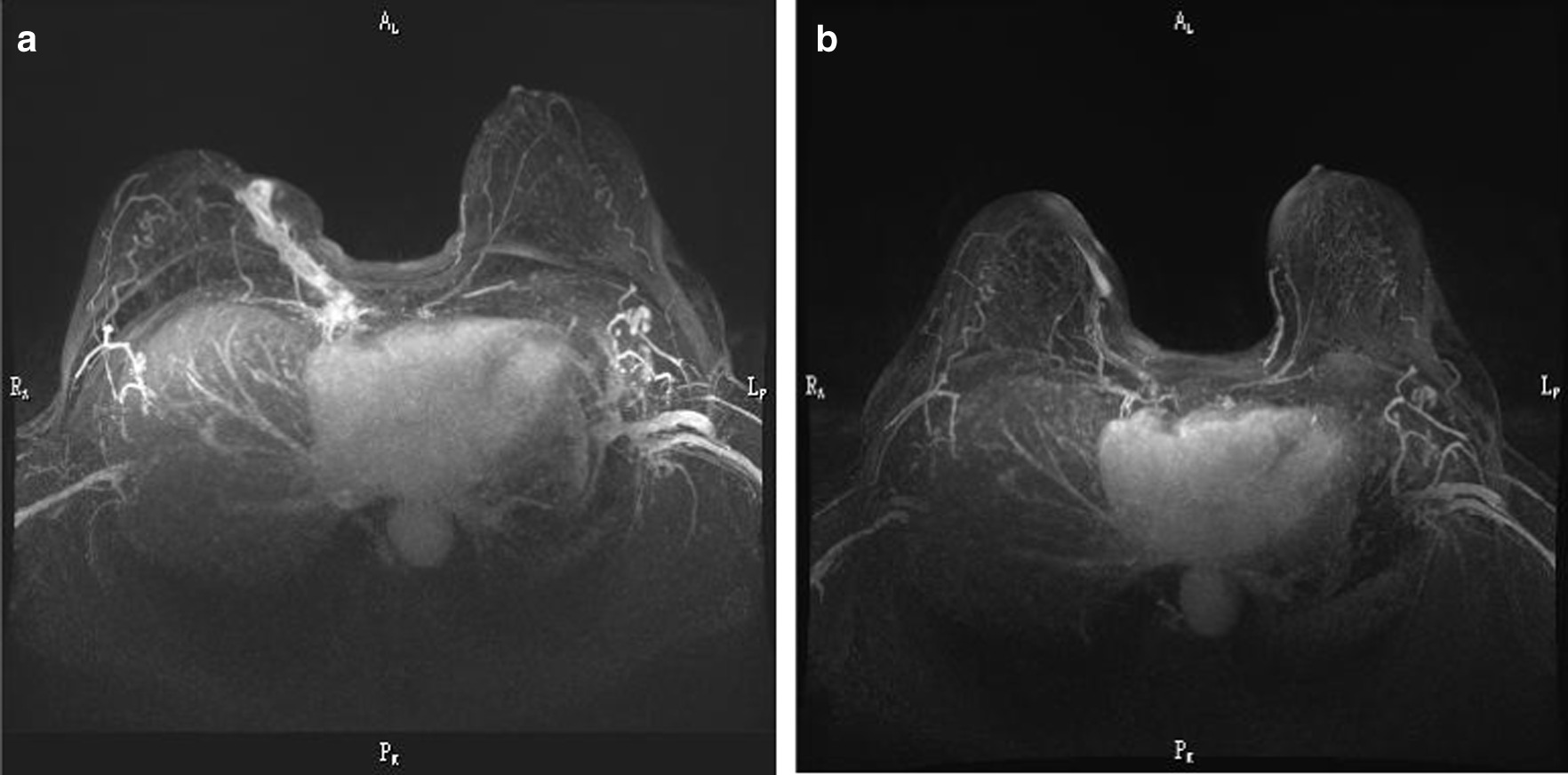
Dynamic subtraction magnetic resonance imaging of the breast. **a** Before treatment: formation of a fistula between the parasternal area and skin of the right breast. **b** After completion of antituberculosis treatment

Mammography showed distortion accompanied by retraction of the right nipple with regional microcalcifications. CT revealed high-density lesions suggesting inflammatory changes in the right breast and calcifications of the right apical pleura and mediastinal lymph nodes. Breast magnetic resonance imaging (MRI) showed no mass in the right mammary gland and a fistula formation between the parasternal area and skin of the right breast (Fig. 2a). T2 imaging showed enhanced high signal intensity lesions in the sternum, suggesting sternal osteomyelitis.

Pus drained from the fistula was collected with a clean syringe and immediately sent to the hospital laboratory to test for aerobic and anaerobic bacteria, mycobacteria, and fungus. Although Gram staining, Ziehl-Neelsen staining, and bacterial and fungal cultures showed negative results, polymerase chain reaction for *M. tuberculosis* was positive. Blood culture was not performed. Four weeks later, the *M. tuberculosis* isolate was cultured from the pus specimen and showed good sensitivity to isoniazid, rifampicin, ethambutol, streptomycin, and levofloxacin. She was diagnosed with tuberculous mastitis and sternal osteomyelitis. A 2-month intensive phase of therapy with isoniazid (300 mg daily), rifampicin (450 mg daily), ethambutol (750 mg daily), and pyrazinamide (1200 mg daily) was initiated, followed by 7-month continuation therapy with isoniazid and rifampicin. After completion of the treatment regimen, the fistula and deformity in the breast were healed (Fig. 1b), and MRI findings of the lesion showed considerable improvement (Fig. 2b). At the 2-year follow-up after treatment, she was cured of the disease and experienced no recurrence or relapse.

## Discussion and conclusions

We experienced a case of refractory mastitis complicated by breast fistula and sternal osteomyelitis, in which a detailed medical history and suspicion of tuberculosis led to the definitive diagnosis and treatment of breast tuberculosis. Breast tuberculosis is a rare condition, accounting for less than 0.1% of all breast diseases and 1.1% of all cases of tuberculosis [3–5]. In endemic countries such as India, tuberculous mastitis has been reported to account for up to 3% of surgically treated breast diseases [6]. Usually, tuberculous involvement of the breast is considered to develop following lymphatic invasion from the parasternal, mediastinal, axillary, or cervical lymph nodes [5, 7]. In addition, hematologic spread from remote lesions or direct extension from the adjacent skin lesions might also occur [4]. Lactation, which involves increased blood flow to the mammary glands, has been reported to be a risk factor for breast tuberculosis [8]. In endemic countries, tuberculous mastitis most commonly occurs in women of reproductive age, that is, those aged 20–40 years [7, 8]. However, as in this case, tuberculous mastitis can occasionally occur in elderly patients [9] and sometimes even in men [7]. In a literature review of 12 cases of tuberculous mastitis between 1984 and 1994 in Japan, the mean age (range) of patients was reported to be 42.8 years (28–84 years) [9].

Tuberculous mastitis often mimics breast cancer because it frequently presents as a firm elastic mass lesion in the unilateral breast [6]. It is usually difficult to distinguish tuberculous mastitis from breast cancer based on ultrasonography and CT imaging [4, 5]. A case of concurrent cancer and tuberculosis in the breast has also been reported. Therefore, clinicians should suspect breast tuberculosis when cytology-nonspecific and/or refractory mass-like lesions are present in the breast.

Although rare, it is important to recognize that tuberculous mastitis can present with refractory abscess or mass lesions that mimic carcinomas in the unilateral breast of women in the reproductive age group and elderly people. The diagnosis of breast tuberculosis might be delayed; hence, it should always be included in the differential diagnoses, particularly in patients with a history of tuberculosis or those living in areas where tuberculosis is endemic.

## Data Availability

Not applicable.

## Abbreviations

CT: Computed tomography
MRI: Magnetic resonance imaging
